# Gonadal hormones and metabolic syndrome in middle-aged and elderly males: results from a prospective cohort study in China

**DOI:** 10.3389/fendo.2024.1365283

**Published:** 2024-06-26

**Authors:** Zhuo Zhang, Yingna Chen, Na Li, Chulin Huang, Diaozhu Lin, Chengzhi Wang, Chunying Wang, Lili You, Lingling Li, Fangping Li, Ying Liang, Huisheng Xiao, Li Yan, Guojuan Lao, Kan Sun

**Affiliations:** ^1^ Department of Endocrinology, Sun Yat-sen Memorial Hospital, Sun Yat-sen University, Guangzhou, China; ^2^ Department of Endocrinology, The Seventh Affiliated Hospital, Sun Yat-sen University, Shenzhen, China

**Keywords:** gonadal hormone, sex hormone binding globulin (SHBG), metabolic syndrome, total testosterone (TT), luteinizing hormone (LH)

## Abstract

**Background:**

Research has shown that gonadal hormones are involved in metabolic pathways relevant to metabolic syndrome (MetS). Nevertheless, no longitudinal study has been conducted on the association between SHBG and MetS in Chinese. The objective of our study was to determine whether there is any association between middle-aged and elderly males in China.

**Methods:**

A total of 531 eligible male subjects, aged above 40 years or older, without MetS at baseline, were recruited. Sex hormone binding globulin (SHBG), total testosterone (TT), follicle-stimulating hormone (FSH), and luteinizing hormone (LH) were measured. A harmonized definition and recommended thresholds for the Chinese population were used to determine metabolic syndrome.

**Results:**

During 3.2 years of follow-up, 20.7% of subjects had developed MetS. Compared with the non-MetS group, subjects in the new-onset MetS group had significantly lower SHBG (43.5 nmol/L [28.8, 74.9] vs 53.7nmol/L [33.8, 115.0], P=0.0018), TT (18.1nmol/L [13.6–21.7] vs 19.5nmol/L[15.0–23.6], P=0.0204), and LH (5.13mIU/L [3.63–7.29] vs 5.87mIU/L [4.05–8.36]) at baseline. The incidence of MetS was decreased according to elevated SHBG quartiles (Q1:26.9%, Q2:22.7%, Q3:21.1%, Q4:12.1%, P for trend =0.0035), TT (Q1: 25.2%, Q2:23.7%, Q3: 17.3%, Q4: 16.7%, P for trend=0.0425), and LH (Q1:25.0%, Q2:21.8%, Q3: 21.8%, Q4: 14.3%, P for trend=0.0411). Compared with those in quartile 4, the OR[CI] of incident MetS for participants in Quartile 1 was 2.33[1.13–4.79] after multiple adjustments. But associations between incident MetS and different quartiles of LH, TT, and FSH were not observed after multiple adjustments. In the subgroup analyses, the significant association between SHBG level and Mets was detected in subjects over 60 years or older, with normal BMI, without insulin resistance, and with eGFR ≥90 mL/min per 1.73m2.

**Conclusion:**

Compared with TT, LH, and FSH, a lower level of SHBG is significantly related to the incidence of MetS among middle-aged and elderly males in China.

## 1 Introduction

Metabolic syndrome(MetS)is defined by a constellation of various metabolic abnormalities, including dyslipidemia, hyperglycemia, elevated blood pressure, and abdominal obesity (1). Powered by advanced technology and standard of living, metabolic syndrome has rapidly increased worldwide (2–9). By 2010, 33.9% of individuals suffered from metabolic syndrome (31.0% of men, 36.8% of women), according to a national epidemiological survey of Chinese adults aged 18 years or older from 31 provinces (4). Mets confer an elevated risk for cardiovascular disease and all-cause mortality (10), leading to an enormous health and economic burden to society. Considering its severe health implications and its high prevalence, there is an imperative need to understand better the factors that drive and influence its pathophysiology.

Recent studies have indicated that lower SHBG and TT levels are significantly associated with an increased risk of developing metabolic disorders (11–20). In a prospective study including 3369 European men, lower baseline TT levels are related to an increased risk of incident MetS, independent of SHBG, BMI or insulin resistance (17). In contrast, Chubb et al. found that lower SHBG was more significantly related to developing metabolic diseases than lower TT in community-dwelling men older than 70 years in Perth, Western Australia (21). And another cross-sectional research found that SHBG was a highly sensitive predictor of MetS in Arab adolescents (22). However, few studies have examined the relationship between serum SHBG levels and MetS in populations of Chinese men. Thus, the main objective of our present study was to explore the associations between SHBG, sex steroids, and incident MetS and investigate which one (SHBG, TT, or other sex steroids) best predicts the development of MetS after adjustment for confounders, using data from a representative and well-characterized cohort of middle-aged and elderly males in China.

## 2 Materials and methods

### 2.1 Subjects

**Figure 1:**
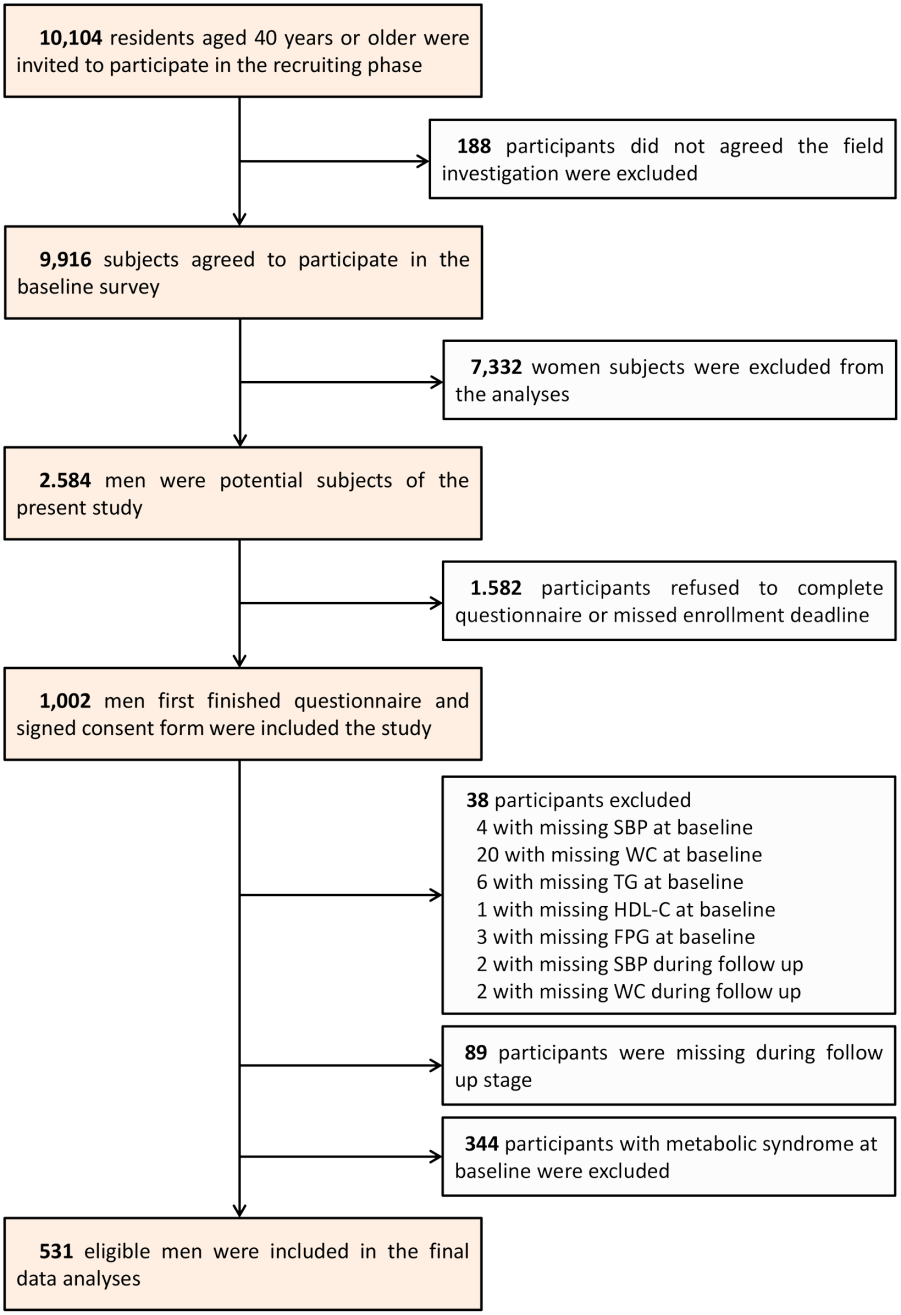
Flowchart of the selection of the study.

We conducted a cohort study in a community from June through November 2011 in Guangzhou, China. Subjects eligible for this study were taken from the Risk Evaluation of Cancers in Chinese Diabetic Individuals: A Longitudinal Study (the REACTION Study). More details of the REACTION Study have been published previously. A total of 10104 subjects aged ≥40 were recruited via home visits or examination notices and 9916 subjects provided written informed consent and enrolled in the project. The participation rate was 98.1%. Firstly, we excluded women from the study, leaving 2584 men in our analyses. Of these men, those who failed to provide information [questionnaire: n=1582; systolic blood pressure (SBP) at baseline: n=4; waist circumference (WC) at baseline: n=20; TG at baseline: n=6; HDL-c at baseline: n=1; fasting plasma glucose at baseline (FPG): n=3; SBP during follow up: n=2; WC during follow up: n=2] were excluded from the study. In addition, 344 subjects with MetS at baseline and 89 subjects who were lost to follow-up were also excluded. Eventually, our analyses included 531 eligible men. The flow chart of the selection of study subjects is shown in **Figure 1**.

### 2.2 Clinical and biochemical measurements

For each participant, personal information was collected via a well-established questionnaire that included demographic characteristics and lifestyle factors. There were three categories of smoking and drinking habits: ‘never’, ‘current’ (having smoked or drunk regularly during the recent half year), or ‘ever’ (having quit smoking or drinking for more than half a year). To estimate the frequency and duration of habitual physical activity, we used a short form of the International Physical Activity Questionnaire (IPAQ). Separate metabolic equivalent hours per week (MET-h/week) were computed to assess the level of physical activity. Standardized anthropometric measurements were completed by staff who had passed the training. Height (accurate to 0.1 cm) and weight (accurate to 0.1 kg) were measured, and body mass index (BMI) was calculated as weight in kilograms divided by height in meters squared (kg/m2). Each participant’s waist circumference (accurate to 0.1 cm) was measured at the umbilical level while standing. Blood pressure was determined as the average of 3 measurements by the same staff at a 5-min interval using a validated automated electronic device (OMRON, Omron Company, China). All blood samples were drawn after overnight fasting for at least 8 hours to measure sex hormone binding globulin (SHBG), total testosterone (TT), luteinizing hormone (LH), follicle-stimulating hormone (FSH), triglyceride (TG), total cholesterol (TC), high-density lipoprotein cholesterol (HDL-C), low-density lipoprotein cholesterol (LDL-C), fasting plasma glucose (FPG), glycosylated hemoglobin (HbA1c), fasting insulin, gamma-glutamyl transferase (γ-GGT), and serum creatinine. The collected venous blood samples were centrifuged at 25°C and stored at -80°C until assay. Considering the possible variation of testosterone and other hormones caused by circadian rhythms, individual blood samples were collected between 7:00 am and 9:00 am.

We used the chemiluminescence techniques to measure TT, LH, and FSH (IMMULITE 2000 Immunoassay System, Siemens Healthcare Diagnostics, USA), and the coefficients of variation were 4.7%–7.5% (intra-assay) and 2.5%–3.3% (inter-assay) for the evaluation of TT. SHBG was assessed by ELISA (DRG International, USA). The reference quartile cutoff points of TT, LH, FSH and SHBG are as follows: TT: [Q1:0–14.6; Q2:14.6–19.2; Q3:19.2–23.5; Q4:>23.5]; LH: [Q1:0–3.97; Q2:3.97–5.71; Q3:5.71–8.06; Q4:>8.06]; FSH: [Q1:0–7.32; Q2:7.32–10.54; Q3:10.54–15.58; Q4:>15.58]; SHBG: [Q1:0–33.1; Q2:33.1–52.2; Q3:52.2–101.1; Q4:>101.1].

An autoanalyzer (ARCHITECT ci16200 Integrated System, Abbott Laboratories, USA) was used for the measurement of TG, TC, HDL-C, LDL-C, FPG, and γ-GGT. HbA1c was determined via the HPLC method (VARIANT II TURBO, Bio-Rad, USA). Insulin was measured by chemiluminescence immunoassay (Centaur XP, SIEMENS, USA). The insulin resistance index (homeostasis model assessment of insulin resistance, HOMA-IR) was calculated using the following equation: HOMA-IR index= [fasting insulin(μIU/ml)] *[fasting glucose (mmol/L)]/22.5. Insulin resistance was estimated by a HOMA-IR index within the top quartile (greater than 2.18 in the present study) (23). Glomerular filtration rate (eGFR) was estimated using the abbreviated Modification of Diet in Renal Disease (MDRD; mL/min/1.73m2) formula: eGFR =186 × [serum creatinine × 0.011]-1.154 × [age]-0.203 × [0.742 if female] ×1.233, where serum creatinine was expressed as mmol/L and 1.233 was the calibration coefficient for Chinese population (24).

### 2.3 Definition of metabolic syndrome

The diagnosis of MetS was based on the harmonized definition of a joint statement of organizations (25). We classified subjects with MetS if they met three or more of the following criteria: (1) raised triglycerides (≥1.7mmol/L) or previous hypertriglyceridemia treatment, (2) reduced HDL cholesterol (<1.0mmol/L in males and <1.3mmol/L in females), (3) high blood pressure (≥130 mmHg systolic and/or ≥85 mmHg diastolic) or under antihypertensive drug treatment, (4) high fasting plasma glucose (FPG ≥5.6mmol/L) or under treatment for glucose-lowering, and (5) central obesity(waist circumference ≥ 85 cm for men or ≥80 cm for women). The cutoff points for waist circumference were determined using Chinese population thresholds.

### 2.4 Statistical analysis

All the participants enrolled were free from MetS at baseline. We divided the study cohort into the non-MetS group and the newly-MetS group, depending on whether MetS was developing or not during the follow-up period. In the data analyses, the data were presented as the means ± SD, medians (interquartile ranges), or numbers (proportions) according to the different variable types. Gonadal hormones were expressed as quartiles, and linear regression models were run to test for trends across groups. Analysis of variance (ANOVA) was adopted for data comparisons among groups. *Post hoc* comparisons were performed using Bonferroni correction when continuous variables that conformed to the normal distribution were analyzed. The Chi-square test was used for the comparison of categorical variables between groups. Unadjusted and multivariate-adjusted logistic regression models were performed to assess the risk of prevalent MetS for each quartile of gonadal hormone levels. Odds ratios (OR) and their corresponding 95% confidence intervals (95% CI) were computed. Model 1 was unadjusted. Model 2 was adjusted for age, BMI, current smoking and drinking status, physical activity level, and SBP. Model 3 was adjusted for age, BMI, current smoking and drinking status, physical activity level, SBP, TG, eGFR, γ-GGT, HbA1c, LDL-C, and HOMA-IR. The relationship between the incidence of MetS and SHBG level was also examined in subgroup analysis and conducted within the age strata (≥ 60/<60years), BMI (normal/overweight and obesity), insulin resistance (yes/no), diabetes (yes/no) and eGFR (≥ 90/< 90ml/min per 1.73 m2). Tests for interaction were conducted by simultaneously including all strata factors, the quartiles of SHBG, and the respective interaction terms (strata factor multiplied by quartiles of SHBG) in the final model 3. All the statistical analyses were carried out with SAS V.9.3 (SAS Institute), and two-sided P-values <0.05 indicated statistical significance.

## 3 Results

### 3.1 Characteristics of the participants

The baseline parameters of the cohort by the MetS status during follow-up were shown in **Table 1**. A proportion of 20.7% of the participants developed MetS with an average follow-up time of 3.2 ± 0.4 years. The mean age of the 531 study subjects was 61.2 ± 7.2 years. Compared with the non-MetS group, subjects in the newly-MetS group had significantly higher BMI, WC, SBP, DBP, TG, TC, LDL-C, FPG, HbA1c, Fasting insulin, HOMA-IR, γ-GGT, and lower HDL-C, TT, LH and SHBG at baseline (all *p*<0.05).

**Table 1:** Characteristics of study population at baseline by incident metabolic syndrome status at follow up.

	Without metabolic syndrome	With metabolic syndrome	P
n (%) ^*^	421 (79.3)	110 (20.7)	< 0.0001
TT (nmol/L)	19.5 (15.0 – 23.6)	18.1 (13.6 – 21.7)	0.0204
LH (mIU/L)	5.87 (4.05 – 8.36)	5.13 (3.63 – 7.29)	0.0340
FSH (mIU/L)	10.54 (7.32 – 15.58)	10.54 (7.34 – 15.14)	0.6298
SHBG (nmol/L)	53.7 (33.8 – 115.0)	43.5 (28.8 – 74.9)	0.0018
Age (years)	58.2 ± 7.3	58.4 ± 6.9	0.7739
BMI (kg/m^2^)	22.3 ± 3.0	24.1 ± 2.3	< 0.0001
WC (cm)	80.3 ± 10.0	84.2 ± 6.9	< 0.0001
SBP (mmHg)	123.1 ± 14.3	128.8 ± 15.2	0.0002
DBP (mmHg)	75.0 ± 9.3	77.7 ± 9.1	0.0075
TG (mmol/L)	1.13 (0.89 – 1.48)	1.39 (1.08 – 1.81)	< 0.0001
TC (mmol/L)	5.04 ± 1.17	5.35 ± 1.06	0.0108
HDL-C (mmol/L)	1.29 ± 0.30	1.22 ± 0.25	0.0131
LDL-C (mmol/L)	3.11 ± 0.90	3.35 ± 0.82	0.0127
FPG (mmol/L)	5.23 (4.94 – 5.58)	5.53 (5.17 – 5.82)	0.0278
HbA1c (%)	5.80 (5.60 – 6.10)	6.00 (5.70 – 6.30)	0.0843
Fasting insulin (μIU/ml)	5.30 (4.00 – 7.40)	7.30 (5.10 – 9.20)	< 0.0001
HOMA-IR	1.24 (0.93 – 1.72)	1.76 (1.27 – 2.36)	< 0.0001
γ-GGT (U/L)	22.0 (17.0 – 30.0)	27.5 (20.0 – 40.0)	< 0.0001
eGFR (ml/min per 1.73 m^2^)	95.8 ± 19.9	95.8 ± 18.4	0.9764
Physical activity (MET-h/week)	21.0 (10.5 – 42.0)	28.5 (18.0 – 56.0)	0.0059
Current smoking [n (%)]	127 (30.7)	30 (27.8)	0.5586
Current drinking [n (%)]	31 (7.6)	8 (7.4)	0.9520

1. Data were means ± SD or medians (interquartile ranges) for skewed variables or numbers (proportions) for categorical variables.

2. ^*^n (%) was for the number of incident metabolic syndrome status at follow up.

3. P values were for the ANOVA or χ^2^ analyses across the groups.

4. TT, total testosterone; FSH, follicle stimulating hormone; LH, luteinizing hormone; SHBG, sex hormone binding globulin; BMI, body mass index; WC, waist circumference; SBP, systolic blood pressure; DBP, diastolic blood pressure; TG, triglycerides; TC, total cholesterol; HDL-C, high-density lipoprotein cholesterol; LDL-C, low-density lipoprotein cholesterol; FPG, fasting plasma glucose; OGTT, oral glucose tolerance test; HOMA-IR, homeostasis model assessment of insulin resistance; eGFR, estimated glomerular filtration rate; γ-GGT, γ-glutamyltransferase.

### 3.2 Associations of gonadal hormones with the incident risk of MetS and its components

**Figure 2:**
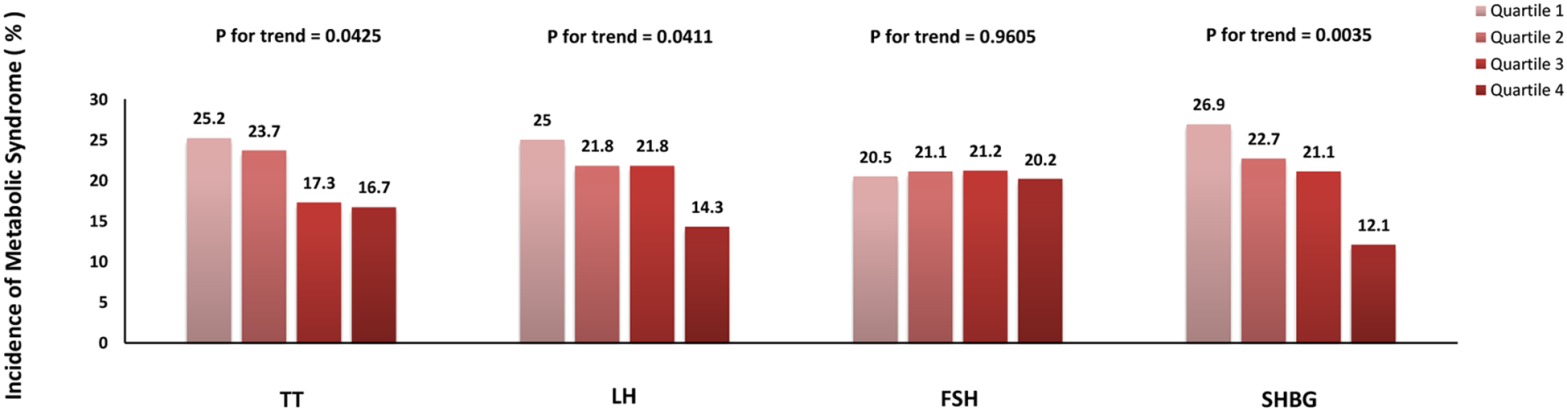
Incidence of metabolic syndrome in different quartiles of gonadal hormones.

The total population was assessed based on the different gonadal hormone levels. As shown in **Figure 2**, the incidence of MetS according to elevated TT quartiles was 25.2%, 23.7%, 17.3% and 16.7% respectively (p for trend = 0.0425), the incidence of MetS was 25.0%, 21.8%, 21.8% and 14.3% respectively (p for trend =0.0411) according to elevated LH quartiles and the incidence of MetS according to elevated SHBG quartiles was 26.9%, 22.7%, 21.1% and 12.1% respectively (p for trend =0.0035). Nevertheless, no obvious trend differences were found according to the elevated FSH quartiles.

Univariate logistic regression analysis showed that subjects in quartile 1 of SHBG had significantly increased odds of incident MetS compared with those in quartile 4 (**Table 2**). After multivariable adjustment for age, BMI, current smoking and drinking status, physical activity level, SBP, TG, eGFR, γ-GGT, HbA1c, LDL-C, and HOMA-IR (Model 3), the OR[CI] of incident MetS for Quartile 1 was 2.33[1.13–4.79] (p<0.05), which suggests that lower SHBG might be considered as a predictor of new-onset MetS and its progression. But there was no statistically significant difference among TT, LH, and FSH (p>0.05).

**Table 2:** Association between gonadal hormones and risk of metabolic syndrome.

		Quartile 1	Quartile 2	Quartile 3	Quartile 4	1-Quartile change of gonadal hormones^*^
TT	Model 1	1.68 (0.92 – 3.08)	1.55 (0.85 – 2.85)	1.05 (0.55 – 1.99)	1	1.22 (1.01 – 1.47)
	Model 2	1.12 (0.58 – 2.17)	1.20 (0.63 – 2.30)	0.99 (0.51 – 1.95)	1	1.05 (0.86 – 1.30)
	Model 3	1.05 (0.53 – 2.10)	1.23 (0.63 – 2.39)	0.99 (0.50 – 1.98)	1	1.04 (0.84 – 1.29)
LH	Model 1	2.00 (1.07 – 3.74)	1.67 (0.89 – 3.16)	1.67 (0.89 – 3.16)	1	1.22 (1.01 – 1.47)
	Model 2	2.13 (1.06 – 4.28)	1.62 (0.80 – 3.28)	1.53 (0.77 – 3.07)	1	1.25 (1.01 – 1.55)
	Model 3	2.03 (0.99 – 4.14)	1.38 (0.67 – 2.85)	1.45 (0.71 – 2.95)	1	1.23 (0.98 – 1.54)
FSH	Model 1	1.02 (0.56 – 1.85)	1.06 (058 – 1.91)	1.07 (0.59 – 1.93)	1	1.01 (0.83 – 1.21)
	Model 2	1.04 (0.52 – 2.06)	0.90 (0.46 – 1.76)	1.01 (0.54 – 1.91)	1	1.00 (0.80 – 1.25)
	Model 3	1.00 (0.49 – 2.04)	0.74 (0.37 – 1.49)	0.92 (0.48 – 1.79)	1	0.98 (0.78 – 1.23)
SHBG	Model 1	2.66 (1.39 – 5.09)	2.13 (1.10 – 4.14)	1.93 (0.99 – 3.77)	1	1.33 (1.10 – 1.61)
	Model 2	2.32 (1.15 – 4.69)	1.88 (0.92 – 3.83)	1.61 (0.79 – 3.30)	1	1.29 (1.04 – 1.60)
	Model 3	2.33 (1.13 – 4.79)	1.74 (0.83 – 3.62)	1.48 (0.71 – 3.10)	1	1.30 (1.04 – 1.62)

Data are odds ratios (95% confidence interval). Participants without metabolic syndrome at follow up are defined as 0 and with metabolic syndrome as 1.

^*^All variables were calculated for 1-Quartile change of decreasing gonadal hormones.

Model 1 is unadjusted.

Model 2 is adjusted for age, BMI, current smoking status, current drinking status and physical activity level.

Model 3 is adjusted for age, BMI, current smoking status, current drinking status, physical activity level, eGFR, γ-GGT, HbA1c, LDL-C and HOMA-IR.

**Figure 3:**
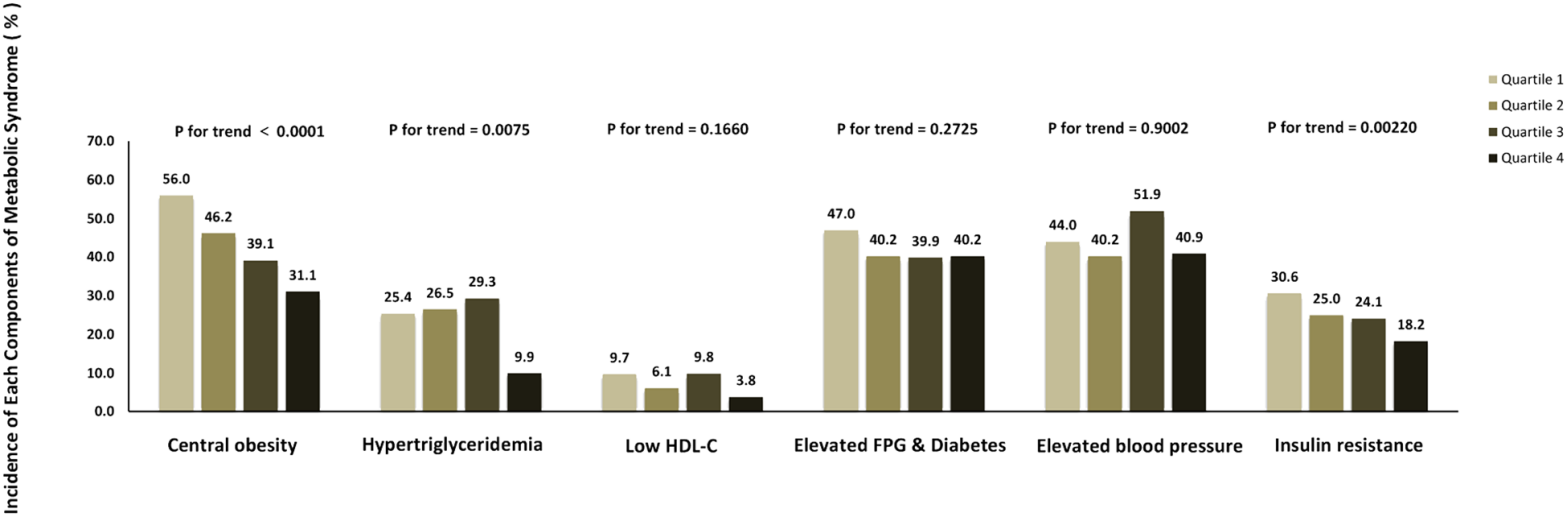
Incidence of each metabolic syndrome components according to different SHBG quartiles.

**Figure 4:**
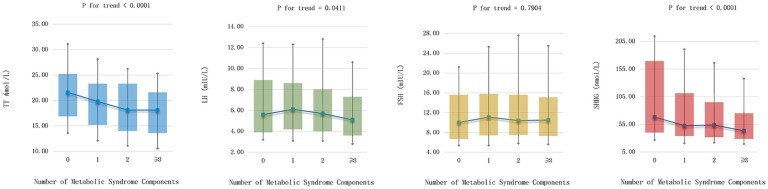
Gonadal hormones levels in different number of metabolic syndrome components.

**Figure 3** shows the incidence of each metabolic syndrome component based on the elevated SHBG quartiles. The incidence rates of hypertriglyceridemia, central obesity, and insulin resistance tended to decrease according to the elevated SHBG quartiles (all p for trend < 0.05). Nevertheless, no significant trend differences were found with low HDL-c, high glucose, and elevated blood pressure. To explore the internal conformance of the above findings, we further analyzed the direct relationship between gonadal hormone levels and the number of metabolic syndrome components. As shown in **Figure 4**, TT, LH, and SHBG levels significantly decreased with the increasing number of metabolic syndrome components (all *p* for trend < 0.05).

### 3.3 The relationship between SHBG level and risk of incident metabolic syndrome in different subgroups

**Table 3** shows the multivariate-adjusted ORs of incident MetS according to decreased SHBG quartiles within different subgroups. The associations between SHBG level and incident MetS were inconsistent in subgroup analyses. Significant relation between SHBG level and Mets was detected in the subjects aged ≥ 60 years, without insulin resistance, with normal BMI, and with eGFR ≥90 mL/min per 1.73m2 (*p* < 0.05).

**Table 3:** Risk of incident metabolic syndrome with each quartile decrease of SHBG levels in different subgroups at follow up.

	n, case/subjects	SHBG (nmol/L)	P^*^	OR 95% CI^#^	P for interaction
Age					0.3756
≥ 60 years	66/299	58.4 (36.6 – 123.6)	–	1.43 (1.08 – 1.89)	
< 60 years	44/232	44.8 (28.9 – 77.0)	< 0.0001	1.12 (0.79 – 1.61)	
Insulin resistance					0.0138
Yes	47/130	44.7 (29.7 – 82.2)	–	0.97 (0.65 – 1.46)	
No	63/401	53.7 (33.8 – 111.7)	0.0122	1.59 (1.20 – 2.11)	
BMI					0.0015
Normal	40/360	55.4 (35.2 – 124.2)	–	1.89 (1.32 – 2.72)	
Overweight and obesity	70/171	45.2 (29.4 – 77.9)	0.0006	0.93 (0.68 – 1.27)	
Diabetes					0.4419
Yes	30/95	49.8 (31.0 – 97.5)	–	1.32 (0.78 – 2.24)	
No	80/436	53.0 (33.3 – 101.3)	0.6532	1.27 (0.98 – 1.64)	
eGFR					
≥ 90 ml/min per 1.73 m^2^	43/203	51.5 (32.2 – 112.1)	–	1.49 (1.03 – 2.16)	0.5655
< 90 ml/min per 1.73 m^2^	67/328	52.2 (33.1 – 97.9)	0.7027	1.30 (0.98 – 1.75)	

Data are odds ratios (95% confidence interval). Participants without metabolic syndrome at follow up are defined as 0 and with metabolic syndrome as 1.

^*^P values were for the ANOVA analyses of SHBG levels in different subgroups.

^#^All variables were calculated for 1-Quartile change of decreasing SHBG. The model is adjusted for age, BMI, current smoking status, current drinking status, physical activity level, eGFR, γ-GGT, HbA1c, LDL-C and HOMA-IR.

## 4 Discussion

The present study evaluated the association between SHBG and metabolic syndrome among middle-aged and elderly males in china. In this study, we found that the serum levels of SHBG, TT, and LH for the MetS group were lower than the non-MetS group at baseline, which is consistent with previous studies. Additionally, SHBG, TT, and LH serum levels were inversely related to incident MetS, which also accords with many previous cross-sectional and longitudinal studies.

Chubb et al. also found a strong inverse association between SHBG level and MetS in both univariate and multivariate analysis (OR was 1.77 (95% CI 1.53–2.06)), based on a large cross-sectional study of 2,502 community-dwelling men aged ≥70 years without diabetes (21). Judith S.Brand et al. conducted a meta-analysis of 20 observational studies, including 12,811 men. They revealed that men with lower concentrations of TT, SHBG, or FT were more likely to have prevalent MetS (ORs per quartile decrease were 1.69 (95% CI 1.60–1.77), 1.73 (95% CI 1.62- 1.85), and 1.46 (95% CI 1.36–1.57) for TT, SHBG, and FT, respectively) and incident MetS (HRs per quartile decrease were 1.25 (95% CI 1.16–1.36), 1.44 (95% 1.30–1.60) and 1.14 (95% 1.01–1.28) for TT, SHBG, and FT, respectively) (26). In addition, longitudinal studies such as the Framingham Heart Study20 (27) of 618 men and the Concord Health and Ageing in Men Project Study21 (28) of 1,705 men showed that only SHBG was independently and significantly associated with incident MetS, neither TT nor FT. And another prospective population-based study of 702 middle-aged men who did not have MetS or T2DM at baseline revealed that men with TT or calculated FT or SHBG levels in the lower quartile had a severalfold increased risk of developing MetS (odds ratio [OR]=2.3, 95% CI 1.5–3.4; 1.7, 1.2–2.5; and 2.8, 1.9–4.1, respectively) after 11 years of follow-up. While there was no significant association between calculated FT with increased risk of MetS after adjusting for potential confounders, including correlates of IR such as BMI, WC, and insulin levels, and including components of MetS such as glucose and triglyceride levels and systolic blood pressure (12), which are similar to our findings. Although there is ample evidence supporting the association between testosterone and MetS, the decrease in testosterone levels is often associated with various factors such as aging, which may confound the onset of MetS. In light of the current situation, whether testosterone supplementation is necessary for MetS treatment remains to be observed and requires conducting randomized controlled trials.

In our study, we found that serum SHBG levels remained inversely associated with the risk of incident MetS after mutual adjustment for confounders such as current smoking and drinking status, physical activity level, age, BMI, SBP, TG, eGFR, γ-GGT, HbA1c, LDL-C, and HOMA-IR, whereas the association between TT, LH and incident MetS was lost. Moreover, the serum SHBG levels significantly decreased with the number of MetS components increasing, indicating that SHBG plays a vital role in the development of MetS. However, some reports were inconsistent with our findings. Therefore, the association between gonadal hormones and the risk of MS or MS characteristics is equivocal.

Moreover, there was still no precise underlying mechanism for how hepatic SHBG influences metabolic components. SHBG is a serum protein produced by hepatocytes, and hepatocyte nuclear transcription factor 4α (HNF-4α) is a transcription factor that regulates the SHBG promoter. HNF-4α levels affect the transcriptional activity and synthesis of SHBG. Monosaccharides reduce HNF-4α levels by directing adipogenesis, reducing SHBG synthesis; therefore, HNF-4α levels are reduced in overweight and obese individuals, suppressing hepatic SHBG expression (29). In addition, the human SHBG promoter contains a peroxisome proliferator-activated receptor response element (PPAR-RE). Peroxisome proliferator-activated receptors (PPARs) are nuclear fatty acid receptors that bind fatty acids and arachidonate, which act as sensors and regulators of lipid and glucose metabolism in many cell types, including hepatocytes. Among the PPARs family members, PPARγ is a significant regulator of adipogenesis and plays a crucial role in hepatic fatty acid accumulation. It was found that PPARγ expression was reduced in SHBG-treated hepatocytes, and inhibition of PPARγ may prevent lipotoxicity, suggesting that SHBG may play a beneficial role in hepatic metabolism by inhibiting adipogenesis. It has been demonstrated that endoplasmic reticulum stress-induced hepatocyte degeneration is closely related to the development of insulin resistance and is accompanied by metabolic syndrome. Katarzyna et al. found that SHBG attenuated palmitate-induced endoplasmic reticulum stress in hepatocytes as well as in the liver of MS patients (30). In addition, lipocalin is closely related to insulin sensitivity, TG, and HDL-C. Decreased lipocalin levels and increased inflammatory factors such as tumor necrosis factor and interleukin 1β can inhibit SHBG production (31, 32) and SHBG may be an early marker of insulin resistance and disorders of glucose and lipid metabolism.

On the other hand, insulin resistance status is associated with low expression of SHBG. Studies have shown that SHBG is not downregulated by insulin in hSHBG transgenic mice, suggesting that insulin has no direct effect on suppressing SHBG (29). Therefore, serum SHBG concentration may be an independent and significant risk factor for metabolic syndrome.

However, there are some limitations to this study. Firstly, our study was based on a single measuring of all gonadal hormones and SHBG, and information on symptoms of hypogonadism and/or gonadotropin levels was lacking. Some participants may have been misclassified due to variability existing in experimental error. Secondly, we did not measure some sex hormones such as androstenedione, progesterone, and DHEAS, so it is impossible to comprehensively evaluate their role in the risk of incident MetS. Thirdly, due to the lack of measurement of free testosterone or bioavailable testosterone in this project, the association between TT and MetS should be interpreted cautiously. Lastly, hormonal and SHBG were also measured by immunoassay, which was less accurate and less reliable than methods such as LC-MS. In addition, because the study only involved Chinese people, the present results cannot be fully generalized to other ethnic groups.

## 5 Conclusions

Our study’s findings suggest an inverse relationship between serum SHBG, TT, and LH levels and the characteristics of MetS. The serum SHBG level, but not serum TT or LH levels, is a dominant and independent risk factor for MetS. Evaluation of gonadal hormones and SHBG may help provide risk stratification strategies or novel therapies to prevent or treat metabolic disorders. Further research should be conducted on the potential molecular mechanisms of how SHBG influences metabolic components in the development of MetS.

## Data availability statement

The raw data supporting the conclusions of this article will be made available by the authors, without undue reservation.

## Ethics statement

The studies involving humans were approved by Ethics Committee of Sun Yat-Sen Memorial Hospital. The studies were conducted in accordance with the local legislation and institutional requirements. The participants provided their written informed consent to participate in this study.

## Author contributions

ZZ: Writing – review & editing, Writing – original draft, Methodology, Investigation, Formal analysis, Data curation. YC: Writing – review & editing. NL: Writing – review & editing. CH: Writing – review & editing. DL: Writing – review & editing. CZW: Writing – review & editing. CYW: Writing – review & editing. LLY: Writing – review & editing, Methodology. LL: Writing – review & editing, Methodology. FL: Writing – review & editing. YL: Writing – review & editing. HX: Writing – review & editing. LY: Writing – review & editing. GL: Writing – review & editing, Supervision. KS: Writing – review & editing, Supervision, Investigation, Funding acquisition, Conceptualization.
